# Predictors of Symptomatic Change and Adherence in Internet-Based Cognitive Behaviour Therapy for Social Anxiety Disorder in Routine Psychiatric Care

**DOI:** 10.1371/journal.pone.0124258

**Published:** 2015-04-20

**Authors:** Samir El Alaoui, Brjánn Ljótsson, Erik Hedman, Viktor Kaldo, Evelyn Andersson, Christian Rück, Gerhard Andersson, Nils Lindefors

**Affiliations:** 1 Department of Clinical Neuroscience, Division of Psychiatry, Karolinska Institutet, Stockholm, Sweden; 2 Department of Clinical Neuroscience, Osher Center for Integrative Medicine, Karolinska Institutet, Stockholm, Sweden; 3 Department of Clinical Neuroscience, Division of Psychology, Karolinska Institutet, Stockholm, Sweden; 4 Department of Behavioural Sciences and Learning, Linköping University, Linköping, Sweden; Macquarie University, AUSTRALIA

## Abstract

**Objective:**

A central goal of health care is to improve patient outcomes. Although several studies have demonstrated the effectiveness of therapist guided internet-based cognitive behaviour therapy (ICBT) for social anxiety disorder (SAD), a significant proportion of patients do not respond to treatment. Consequently, the aim of this study was to identify individual characteristics and treatment program related factors that could help clinicians predict treatment outcomes and adherence for individuals with SAD.

**Method:**

The sample comprised longitudinal data collected during a 4-year period of adult individuals (N = 764) treated for SAD at a public service psychiatric clinic. Weekly self-rated Liebowitz Social Anxiety Scale (LSAS-SR) scores were provided. Rates of symptomatic change during treatment and adherence levels were analysed using multilevel modelling. The following domains of prognostic variables were examined: (a) socio-demographic variables; (b) clinical characteristics; (c) family history of mental illness; and (d) treatment-related factors.

**Results:**

Higher treatment credibility and adherence predicted a faster rate of improvement during treatment, whereas higher overall functioning level evidenced a slower rate of improvement. Treatment credibility was the strongest predictor of greater adherence. Having a family history of SAD-like symptoms was also associated with greater adherence, whereas Attention-Deficit/Hyperactivity Disorder (ADHD)-like symptoms, male gender, and family history of minor depression predicted lower adherence. Also, the amount of therapist time spent per treatment module was negatively associated with adherence.

**Conclusions:**

Results from a large clinical sample indicate that the credibility of ICBT is the strongest prognostic factor explaining individual differences in both adherence level and symptomatic improvement. Early screening of ADHD-like symptoms may help clinicians identify patients who might need extra support or an adjusted treatment. Therapist behaviours that promote adherence may be important for treatment response, although more research is needed in order to determine what type of support would be most beneficial.

## Introduction

Social anxiety disorder (SAD) is one of the most common anxiety disorders [1] and is often described as following a chronic course if untreated. SAD is characterized by an intense fear in social situations and is associated with significant costs, both for the affected individuals and for society [2]. The effectiveness of cognitive behavioural therapy (CBT) in the treatment of SAD is well established [3]. To increase accessibility to CBT, guided internet-based CBT (ICBT) has been developed with outcomes comparable with face-to-face CBT [4]. However, a significant proportion of patients do not achieve clinically significant improvement, irrespective of treatment modality. For example, it has been reported that up to 50% of patients do not respond to first-line treatments such as CBT or pharmacotherapy [5]. Also, in a study on ICBT, 23% of subjects did not improve on any measure [6]. When ICBT is considered for dissemination within regular clinical practice, identifying prognostic factors that explain individual differences in how patients respond to ICBT when administered within the context of routine care could lead to improvements in treatment delivery. Specifically, this may help clinicians better predict how different subgroups of patients with SAD are likely to respond to ICBT. Such knowledge could contribute to treatment modifications that decrease outcome variability and increase treatment effectiveness for a wider range of patients.

There are few studies on outcome predictors of ICBT for SAD and little consensus regarding the evidence of these. This might be due to several reasons such as differences in samples size, sample characteristics and statistical methods for analysing the prognostic value of baseline variables. Therefore, our selection of outcome predictors was not restricted to internet interventions but refers to the larger literature of CBT for SAD [7,8] and is guided by previous research in this area [9,10]. In line with a number of recent outcome prediction studies on SAD [11–14] and constrained by available patient data routinely collected within the clinic, variables with potential prognostic value were categorized into four domains: (1) socio-demographic variables (e.g. age, gender), (2) clinical characteristics (e.g. illness severity, comorbidity), (3) family history of mental illness and (4) treatment-related factors (e.g. adherence, treatment credibility and amount of therapist support).

Examples of identified outcome predictors within the socio-demographic domain that have been found to be associated with a positive outcome in ICBT for SAD are employment status (i.e. working full-time) and having children [13]. In regard to clinical characteristics, illness severity and number of comorbid diagnosis have also been identified as significant outcome predictors [15]. Also, although comorbid depression has been related to lower treatment response during both CBT [16,17] and ICBT [13] for SAD and higher illness severity after treatment [17,18], several studies have not been able to link comorbid depression to the rate of symptomatic change during treatment (e.g. [18,19]), and a recent study found no predictive value of comorbid depression [15]. In regard to treatment-related factors, treatment credibility and adherence have been identified as being of special relevance to the effectiveness of self-help interventions such as ICBT [20,21]. Several studies have found that credibility of ICBT [13,14] and adherence to either face-to-face CBT or ICBT [6,13,14,22,23] is associated with treatment outcome. However, since adherence is measured during or after treatment completion, it may be of limited clinical use as a pre-treatment prognostic predictor. Therefore, examining adherence not only as an outcome predictor but also as an outcome itself has been identified as an aspect of prediction research on ICBT that needs more attention [24]. We are only aware of one study that has examined this relationship in regard to ICBT for SAD specifically, where treatment credibility was found to predict adherence in unguided self-help, but not in guided self-help.

The aim of this study was to examine individual differences in response to ICBT for SAD and to facilitate possible future identification of patients who might need additional support during treatment. To identify predictors of symptomatic change and adherence of ICBT within the context of a routine clinical setting, we explored effectiveness data obtained at a publicly funded psychiatric clinic specialized in delivering ICBT. Based on findings in previous research on ICBT, we hypothesized that treatment credibility and adherence would have prognostic value also in a naturalistic setting, where higher levels of these factors would likely be associated with a more favourable outcome. To analyse the data, multilevel modelling techniques were used. These statistical methods take advantage of all available data collected during the study period and have been shown to produce more precise parameter estimates and require fewer statistical assumptions [25,26]. A stepwise procedure adopted by Fournier et al. [27] was followed where predictor domains (socio-demographic variables, clinical variables, family history and treatment factors) were first examined separately. This allowed us to determine the predictive value of each variable while controlling for the other variables within each domain. Then, a final model was tested where only those variables identified as significant outcome predictors within each domain were simultaneously included.

## Materials and Methods

### Participants

The study sample consisted of 764 adult patients, all of which had a principal diagnosis of SAD and had undergone treatment as part of routine outpatient psychiatric care at a specialized public ICBT clinic at the time of data extraction (i.e. patients having completed treatment between October 2010 and October 2014). Patients were admitted to treatment either through self-referral or through referral from psychiatric or primary care. All patients first completed an on-line screening battery of self-report questionnaires. Then, clinician administered assessments were conducted before admittance where the Mini-International Neuropsychiatric Interview (MINI) [28] diagnostic interview was used by psychiatrists (or resident physicians supervised by psychiatrists) to diagnose Axis I DSM-IV disorders.

### Ethics Statement

The study, including its consent procedure, was approved by the Regional Ethical Review Board in Stockholm, Sweden (no 2011/2091-31/3). Since this research was conducted as a retrospective cohort study, active informed consent was not required and therefore not obtained. However, as required by the ethics committee, a letter explaining the study was sent to all participants prior to data collection providing a choice of participation. Thus, participation in the study was obtained through an opt-out methodology. This approach (i.e. passive consent) is considered to be the most efficient procedure for registry data that does not violate the option of providing choice [29] and also increases the likelihood of having a more representative sample [30].

### Study Design and Treatment

Over the study period, approximately 25 different psychologists delivered the treatments. These were trained in CBT and either licensed or occasionally resident psychologists under clinical supervision. Therapists followed a treatment manual originally developed by Andersson and colleagues [31] with documented effects in several randomized controlled trials [32] adhering to Clark and Wells’ [33] cognitive model of social phobia developed for individual therapy of SAD. Non-inferiority of the treatment compared to conventional group-based CBT has previously been documented within the context of routine psychiatric care [34].

Treatment modules consisted of psycho-educative text and supporting figures, complemented with homework assignments. This is similar to self-help books used in bibliotherapy, with the difference that the material was accessed online after logging in to a secure treatment website. In ICBT, treatment modules are the equivalence of sessions in conventional CBT. However, since the treatment content was presented in text form, patients were able to learn at their own convenience. Similar to sessions in CBT, modules were administered sequentially; after completion of a module and its homework assignment, the subsequent module was activated by the therapist. This meant that patients had to complete a module in order to get access to the following module.

The first module introduced patients to CBT for SAD providing rationale for the treatment. This was followed by exercises in cognitive restructuring to help patients understand how SAD is maintained and learn how to recognize maladaptive or negative automatic thoughts with the aid of a detailed review of Clark & Wells’ cognitive model. Patients were also taught how to challenge negative thoughts and encouraged to reflect on treatment goals. In the next phase of treatment, behavioural experiments were introduced, including gradual exposure to feared situations. During these modules, patients learned about the function of safety behaviours and experimented with attentional refocussing to enhance the effect of exposure. The final modules contained material on developing communication and social skills and relapse prevention strategies. The last module encouraged patients to review what they had learned throughout the treatment, and to make a plan for continued improvements and how to manage setbacks.

The role of the therapists was to guide patients through each treatment module. This was conducted through online written conversations within the secure treatment platform. This included not only providing feedback on homework assignments, but prompting patients when they were inactive or helping them with problem solving when experiencing difficulties. Thus, more therapist time tended to be allocated to patients having a greater need for support and guidance.

### Outcome Measures

The primary outcome measure was the self-rated version of the Liebowitz Social Anxiety Scale, LSAS-SR [35]. The LSAS-SR has reported strong psychometric properties with an internal consistency of *α* =. 95 and a 12-week test-retest reliability of *r* =. 83 [35,36]. The LSAS has also been evaluated for internet administration with a reported internal consistency of *α* =. 94 [37]. For the current sample, Cronbach's alpha of the LSAS-SR at pre-treatment was. 95. Besides at pre- and post-treatment, social anxiety was measured weekly throughout the active treatment phase. The secondary outcome measure was treatment adherence, operationalized as the number of activated modules.

### Potential Predictor Variables

All potential predictors of symptomatic change and adherence were measured at baseline except for treatment program factors, which were measured during treatment (e.g. treatment credibility was assessed during the second week) or after treatment (e.g. adherence). Each variable was assigned to one of the following four domains: socio-demographic variables, clinical characteristics, family history of mental illness and treatment-related factors.

#### Socio-demographic variables

This domain comprised 6 variables: age, gender, level of education, employment status (dichotomized; i.e. working part- or full-time or student vs. unemployed, part- or full-time sick leave or disability retirement), cohabitation status (dichotomized; i.e. single, divorced, widowed, or separated vs. married or living with partner) and having children.

#### Clinical characteristics

The clinical domain consisted of 14 variables: Clinical Global Impression—Severity Scale [38] which was rated on a 7-point scale; co-morbid depressive symptoms were measured at baseline using the self-rated Montgomery-Åsberg Depression Rating Scale (MADRS-S) [39]; the Alcohol Use Disorders Identification Test (AUDIT) [40] was used prior to treatment to screen for alcohol problems and the Drug Use Disorders Identification Test (DUDIT) [41] was used to screen for drug misuse; the Adult ADHD Self-Report Scale-V1.1 (ASRS) was used as a short screening instrument for adult ADHD-like symptoms [42]; the number of co-morbid diagnoses was assessed during the clinician diagnostic interview using the MINI; the Global Assessment of Functioning (GAF) was used to assess social, occupational, and psychological functioning; years since onset of symptoms was assessed during the clinical interview; age of onset of symptoms was calculated by subtracting the reported number of years since onset of symptoms from the patient’s age at the time of treatment; generalized beliefs of self-efficacy was assessed using the General Self-Efficacy Scale (GSES) [43]; concurrent psychotropic medication, history of depression, history of inpatient psychiatric care and history of attempted suicide were assessed during the diagnostic interview.

#### Family history of mental illness

The family history domain consisted of 14 variables reporting occurrence of family history of mental illness: family history of social anxiety disorder, social anxiety disorder-like symptoms, anxiety, depression, minor depression, panic disorder, neuropsychiatric condition, psychosis, bipolar disorder, dependence / substance abuse, suicide attempts and family history of completed suicide. These were assessed during the diagnostic interview by a clinician prior to treatment as part of the intake assessment process at the clinic in the form of a checklist. As such, the measure of family history was created for this study as a categorical variable.

#### Treatment-related factors

The treatment domain consisted of 9 variables: adherence, operationalized as the number of activated treatment modules; treatment credibility, operationalized as the total score of the credibility/expectancy scale [44] where patients’ attitudes to the credibility of the treatment and expectancy regarding treatment effectiveness were rated on a 10-point scale (0 = not at all to 10 = very much) after the first week of the treatment; therapist time (hours); therapist time per module (minutes); patients’ logged time online; patients’ number of logins; patients’ number of mouse clicks; patients’ number of sent messages and patients’ number of posted messages on a common discussion forum within the treatment platform.

### Data Analysis

#### Multilevel model of symptomatic change

When analysing predictors of symptomatic improvement during the course of treatment, a longitudinal multilevel framework was applied using the linear mixed-effects models procedure in SPSS version 21. All available data was used in a full intent-to-treat analysis approach with full maximum likelihood estimation procedures for all multilevel models. The objective of using multilevel modelling was to model change in social anxiety over the treatment period and to examine the influence of predictors on this trajectory. This was studied by testing the interaction effect of each predictor × time product; i.e. whether the effect of time on social anxiety varied depending on the value of the predictor variable.

Due to the relatively large number of potential predictor variables, these were grouped into the four domains described above. As such, all variables were first tested in separate models before building a final model of change that included significant variables from all domains. Each domain of predictors were explored in a stepwise manner following the procedure adopted by Fournier et al. [27] and Amir et al. [11]. In Step 1, each model therefore included all variables of that domain, which were then screened in regard to their significance levels. Since the main interest of the study was in the factors that have an effect on the rate of symptomatic improvement, potential prognostic variables were required to have a significant interaction with time in order to progress to the next step. However, each consecutive model still included both the main effect (i.e. the effect on post-treatment level of social anxiety) of each added predictor as well as its interaction with time (i.e. the effect on the rate of change). Those that were found to be significant at *p* <. 2 in Step 1 were tested again in a second model (Step 2). Increasingly stricter significance levels were applied at each step and this screening procedure was repeated at *p* <. 10 (Step 3) and at *p* <. 05 (Step 4). Only those variables that remained significant at Step 4 (*p* <. 05) within each domain were included in a final model where the effect of each predictor was tested while simultaneously controlling for the effects of the others.

Each multilevel model consisted of two levels, where Level 1 was the repeated measurements describing the linear change of social anxiety within patients over the course of treatment (a quadric change trajectory was also tested, but because it did not significantly describe the shape of change over time it was not included in the models) and Level 2 consisted of the patients with their socio-demographic, family history, clinical and treatment-related characteristics. In line with the approach described by Smits and colleagues [12], the time variable was re-centred at post-treatment; estimates of the intercept therefore reflect the predicted level of social anxiety at post-treatment. We also chose to recode the time variable between -1and 0 as proposed by Heck et al. [45]. This coding has the benefit that the estimated coefficients reflect the magnitude of change in social anxiety achieved over the entire treatment period (as opposed to the amount of weekly change). Therefore, since there were 12 measurement occasions, the distance between each step was 0.083; the time variable was therefore coded as -1, -0.92, -0.83, -0.67, -0.58, -0.50, -0.42, -0.33, -0.25, -0.17, -0.08 and 0. Finally, to facilitate comparison of coefficients for predictors measured on different scales, we followed the approach by Smits and colleagues [12] where predictor variables were standardized into *z*-scores prior to analysis.

#### Analysing adherence as outcome

In the case of analysing adherence as an outcome, selection of variables to enter into the mixed-effects regression model was based on the same stepwise procedure described above. However, in the treatment factors domain, only treatment credibility and therapist time per module were tested since patient activity such as number of logins, mouse clicks, messages and time online were variables highly correlated with adherence (*p* <. 001).

## Results

### Descriptive Statistics of the Data

Descriptive patient characteristics are presented in Tables 1–4. Means for the main outcome measure, standard deviations and missing data for each measurement occasion are presented in Table 5.

**Table 1:** Descriptive statistics of examined predictor variables within the socio-demographic domain.

					95% Confidence Interval	
	N	Mean	Std. Deviation	Std. Error Mean	Lower	Upper
Socio-demographic variables						
Age (years)	764	32.51	8.98	0.33	31.88	33.15
Gender (% women)^a^	764	46%	0.50	0.02	0.42	0.49
Level of education^b^	726	5.77	1.33	0.05	5.68	5.87
Employment^a^	729	85%	0.35	0.01	0.83	0.88
Married / cohabiting^a^	726	54%	0.50	0.02	0.50	0.58
Have children^a^	729	32%	0.47	0.02	0.29	0.35

^a^ Valid percent is reported, i.e. the rate of those who provided data.

^b^ Level of education was rated on a 7-point scale (1 = less than 7–9 years in school; 2 = 7–9 years in school; 3 = incomplete vocational or secondary school; 4 = vocational school; 5 = secondary school; 6 = university, started but not completed studies; 7 = completed university studies).

**Table 2:** Descriptive statistics of examined predictor variables within the family history of mental illness domain.

					95% Confidence Interval	
	N	Mean	Std. Deviation	Std. Error Mean	Lower	Upper
Family history of mental illness						
Family history of social anxiety disorder^a^	729	5%	0.22	0.01	0.04	0.07
Family history of social anxiety disorder-like symptoms^a^	729	20%	0.40	0.02	0.17	0.23
Family history of anxiety^a^	729	7%	0.26	0.01	0.05	0.09
Family history of depression^a^	729	18%	0.39	0.01	0.15	0.21
Family history of minor depression^a^	729	9%	0.29	0.01	0.07	0.11
Family history of panic disorder^a^	729	3%	0.16	0.01	0.02	0.04
Family history of neuropsychiatric condition^a^	729	4%	0.20	0.01	0.03	0.06
Family history of psychosis^a^	729	3%	0.18	0.01	0.02	0.05
Family history of bipolar disorder^a^	729	6%	0.23	0.01	0.04	0.07
Family history of dependence / substance abuse^a^	729	13%	0.34	0.01	0.11	0.16
Family history of suicide attempts^a^	729	4%	0.20	0.01	0.03	0.06
Family history of suicide completed^a^	729	5%	0.23	0.01	0.04	0.07

^a^ Valid percent is reported, i.e. the rate of those who provided data.

**Table 3:** Descriptive statistics of examined predictor variables within the clinical characteristics domain.

					95% Confidence Interval	
	N	Mean	Std. Deviation	Std. Error Mean	Lower	Upper
Clinical characteristics						
Baseline LSAS-SR (self-rated)	764	71.23	24.57	0.89	69.48	72.97
Baseline LSAS (clinician rated)	690	68.16	23.04	0.88	66.44	69.88
CGI-S	708	3.79	0.83	0.03	3.73	3.85
GAF	606	61.33	7.78	0.32	60.71	61.95
Comorbidity^a^	729	29%	0.57	0.02	0.25	0.34
Comorbid panic disorder^a^	729	5%	0.21	0.01	0.03	0.06
Comorbid agoraphobia^a^	729	4%	0.20	0.01	0.03	0.06
Comorbid mild depressive episode^a^	729	4%	0.20	0.01	0.03	0.05
Comorbid moderate depressive episode^a^	729	3%	0.18	0.01	0.02	0.04
Comorbid severe depressive episode^a^	729	0%	0.00	0.00	-	-
Comorbid recurrent mild depressive episode^a^	729	4%	0.19	0.01	0.02	0.05
Comorbid recurrent moderate depressive episode^a^	729	5%	0.21	0.01	0.03	0.06
Comorbid recurrent severe depressive episode^a^	729	0%	0.04	0.00	0.00	0.00
Comorbid recurrent depression without current symptoms^a^	729	4%	0.20	0.01	0.03	0.05
Comorbid dysthymia^a^	729	1%	0.10	0.00	0.00	0.02
MADRS-S	745	14.70	7.81	0.29	14.14	15.26
ASRS	386	2.57	1.76	0.09	2.39	2.75
AUDIT	726	5.60	4.96	0.18	5.24	5.97
DUDIT	726	0.57	2.67	0.10	0.37	0.76
Age of onset of symptoms	660	16.52	8.44	0.33	15.88	17.17
Years since onset of symptoms	660	15.95	10.99	0.43	15.11	16.79
Concurrent psychotropic medication	378	68%	0.47	0.02	0.64	0.73
General self-efficacy	634	26.33	5.52	0.22	25.90	26.76
History of inpatient psychiatric care^a^	729	4%	0.20	0.01	0.03	0.06
History of depression^a^	729	48%	0.50	0.02	0.45	0.52
Attempted suicide^a^	729	6%	0.24	0.01	0.04	0.08

Note. LSAS-SR; Liebowitz Social Anxiety Disorder Scale—self-rated. MADRS-S; Montgomery–Asberg Depression Rating Scale score—self-rated. ASRS; Adult ADHD Self-Report Scale. DUDIT; Drug Use Disorder Identification Test. AUDIT; Alcohol Use Disorders Identification Test. CGI-S; The Clinical Global Impression—Severity scale.

^a^ Valid percent is reported, i.e. the rate of those who provided data.

**Table 4:** Descriptive statistics of examined predictor variables within the treatment-related factors domain.

					95% Confidence Interval	
	N	Mean	Std. Deviation	Std. Error Mean	Lower	Upper
Treatment-related factors						
Adherence^a^	764	7.72	3.36	0.12	7.48	7.96
Treatment credibility	633	36.20	7.42	0.30	35.62	36.78
Therapist time per module (minutes)	764	20.58	11.24	0.41	19.78	21.38
Therapist time (hours)	764	2.52	1.51	0.05	2.41	2.63
Patient logged time online (hours)	764	33.27	32.48	1.17	30.96	35.58
Patient number of logins	764	41.50	26.67	0.97	39.61	43.39
Patient number of mouse clicks	764	795.44	496.58	17.97	760.17	830.71
Patient number of sent messages	764	14.78	8.16	0.30	14.20	15.36
Patient number of posted messages on forum	764	1.81	4.49	0.16	1.49	2.13

^a^ Adherence was operationalized as the number of activated treatment modules.

**Table 5:** LSAS-SR means and missing data for each measurement occasion.

Time	N (%)	Missing (%)	Mean	Std. Deviation
**Pre-treatment**	764 (100%)	0 (0%)	71.23	24.57
**Week 1**	674 (88%)	90 (12%)	69.27	24.66
**Week 2**	687 (90%)	77 (10%)	67.04	25.10
**Week 3**	668 (87%)	96 (13%)	64.96	25.04
**Week 4**	659 (86%)	105 (14%)	63.46	25.17
**Week 5**	613 (80%)	151 (20%)	61.41	25.60
**Week 6**	582 (76%)	182 (24%)	59.65	25.84
**Week 7**	571 (75%)	193 (25%)	57.83	25.14
**Week 8**	546 (71%)	218 (29%)	56.34	25.91
**Week 9**	536 (70%)	228 (30%)	53.68	25.32
**Week 10**	502 (66%)	262 (34%)	51.57	25.17
**Post-treatment**	657 (86%)	107 (14%)	50.57	25.40

Since the study mainly relies on self-reported outcomes, a bivariate correlation analysis was performed to assess the relationship between self-reported and clinician-rated measures. Self-rated and clinician-rated LSAS scores were highly correlated; *r* = 0.86, *n* = 690, *p* < 0.001, although a dependent-samples t-test detected a significant difference between the clinician-rated scores (*M* = 68.16, *SD* = 23.04) and self-rated scores (*M* = 70.75, *SD* = 24.20); *t*(689) = 5.32, *p* <.001, suggesting that patient ratings tend to be systematically higher than clinician ratings.

### Stepwise Analyses

Results of the stepwise analyses for each domain exploring predictors of symptomatic change are provided in S1 Table. S2 Table presents the results of these procedures for predicting adherence levels.

#### Socio-demographic variables

In the final step for the socio-demographic domain, having a higher age and being employed were both associated with a slower rate of improvement in social anxiety, although these factors were also associated with lower post-treatment LSAS-SR scores (see S1 Table). In other words, these change trajectories were lower and flatter when compared with younger or unemployed subjects. Regarding predictors of treatment adherence, male gender was associated with lower adherence, whereas the level of education was positively associated with adherence (see S2 Table).

#### Family history of mental illness

In the final step for the family history domain, none of the conditions were related to the rate of symptomatic improvement or post-treatment LSAS-SR scores (see S1 Table). However, three of the conditions in this domain were significantly associated with adherence; having a family history of social anxiety predicted a higher level of adherence, whereas having a family history of depression or minor depression predicted lower adherence (see S2 Table).

#### Clinical characteristics

In the final step of the clinical characteristics domain, only GAF had a significant effect on the rate of improvement, where having a higher level of functioning was associated with slower improvement (see S1 Table). GAF was also associated with a lower post-treatment LSAS-SR score, indicative of a lower and flatter change trajectory. Finally, having more ADHD-like symptoms (higher ASRS scores) was associated with lower adherence (see S2 Table).

#### Treatment-related factors

In the final step for the treatment-related factors domain, higher adherence and higher perceived treatment credibility both predicted a faster rate of improvement and less social anxiety after treatment (see S1 Table).

In the final step in predicting adherence, treatment credibility predicted a higher level of adherence, whereas a higher amount of therapist time spent per module was associated with lower levels of adherence (see S2 Table).

### Final Models Including All Significant Prognostic Variables

The final multilevel models predicting rate of improvement and level of adherence included simultaneous entry of those significant prognostic variables that had been identified in the stepwise analyses of each domain. As such, the effect of each variable was evaluated while controlling for the effects of the other variables.

#### Predictors of symptomatic change

**Fig 1:**
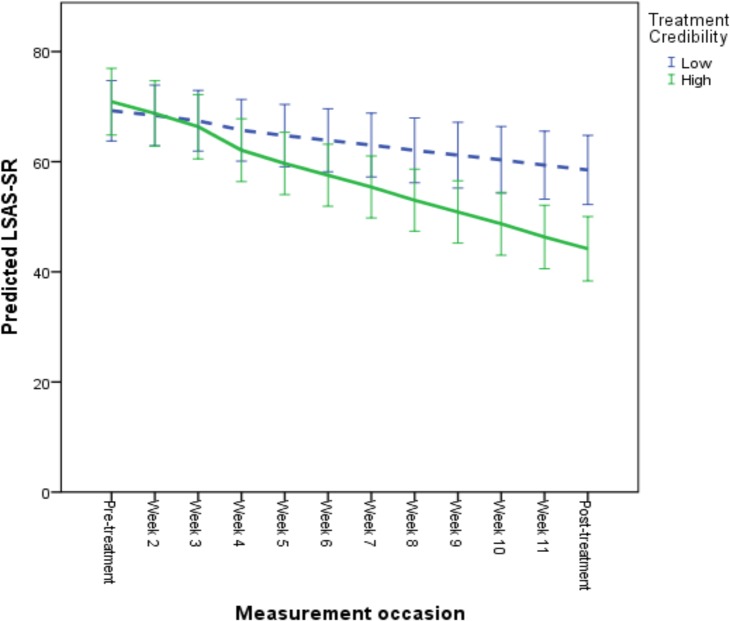
Predicted symptomatic improvement based on individual differences in perceived treatment credibility. For illustrative purposes, a categorization was performed to depict predicted growth curves for patients scoring high and low on credibility. High credibility was operationalized as 1 standard deviation above the mean score on the treatment credibility scale and low credibility as 1 standard deviation below the mean. Mean treatment credibility was 36.20 (SD = 7.42). Error bars are displayed with 95% confidence intervals.

**Fig 2:**
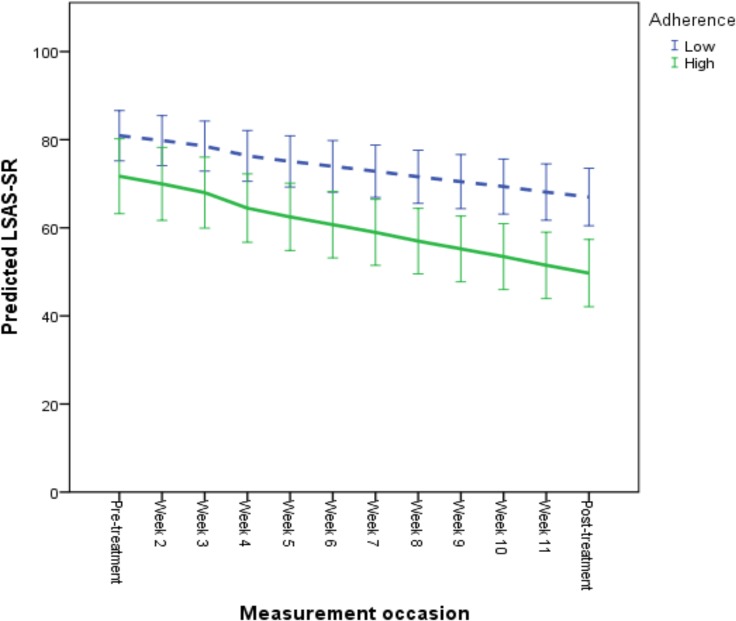
Predicted symptomatic improvement based on individual differences in treatment adherence. For illustrative purposes, a categorization was performed to depict predicted growth curves for patients with high and low adherence. High adherence was operationalized as 1 standard deviation above the mean activated number of treatment modules and low adherence as 1 standard deviation below the mean number of modules. Mean adherence was 7.72 (SD = 3.36). Error bars are displayed with 95% confidence intervals.

**Fig 3:**
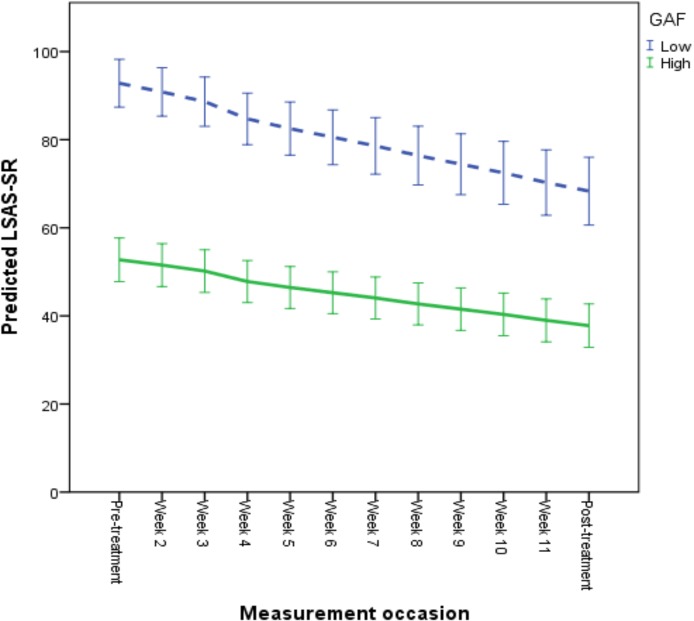
Predicted symptomatic improvement based on individual differences in overall functioning level. Overall level of functioning was as measured with the Global Assessment of Functioning (GAF). For illustrative purposes, a categorization was performed to depict predicted growth curves for patients with high and low levels of functioning. High GAF was operationalized as 1 standard deviation above the mean and low GAF as 1 standard deviation below the mean. Mean GAF was 61.33 (SD = 7.78). Error bars are displayed with 95% confidence intervals.

The results of the longitudinal growth model predicting symptomatic improvement are presented in Table 6 and in Figs 1, 2 and 3. For illustrative purposes, a categorization was performed to depict predicted growth curves for patients scoring high and low on each predictor variable, where a “high” score was operationalized as one standard deviation above the mean and a “low” score as one standard deviation below the mean. According to the final model, age and employment status did not have an effect on improvement when controlling for patients’ overall functioning level, treatment credibility ratings and adherence levels. Specifically, a high level of perceived treatment credibility was associated with a faster rate of improvement and lower social anxiety after treatment (see Fig 1). Also, a high level of adherence was associated with a greater improvement rate and lower post-treatment LSAS-SR scores (see Fig 2). Patients with a higher overall functioning level also evidenced lower post-treatment LSAS-SR scores, although these individuals had a slower rate of improvement (see Fig 3).

**Table 6:** Final model of predictors’ influence on the rate of symptomatic change and post-treatment level of social anxiety.

	Change over time		Post-treatment score	
	*b* (SE)^a^	*p*	*b* (SE)^b^	*p*
**Outcome**				
Social anxiety (LSAS-SR)	-19.00 (.90)	***	51.97 (1.10)	***
**Predictor**				
Age	1.34 (1.12)		-1.39 (.91)	
Employment	.46 (1.16)		-2.29 (.95)	*
GAF	3.35 (1.16)	***	-7.97 (.95)	***
Adherence	-4.02 (1.26)	***	-5.95 (1.04)	***
Treatment credibility	-4.15 (1.14)	***	-3.06 (.93)	**

Note. Significant effects on outcome are denoted as:

***, *p* < ∙001;

**, *p* < ∙01 and

*, *p* < ∙05.

^a^ Values represent standardized beta coefficients predicting the rate of change (slope) in self-rated Liebowitz Social Anxiety Scale (LSAS-SR) scores over assessment occasions. Coefficients therefore represent the interaction of the predictor with time. Negative values indicate greater change during treatment.

^b^ Values represent standardized beta coefficients predicting LSAS-SR at post-treatment. Negative values indicate lower estimated post-treatment scores.

#### Predictors of treatment adherence

Results from the model predicting adherence are presented in Table 7. Specifically, a high level of treatment credibility was associated with higher adherence. Also, those patients who had a family history of SAD-like symptoms evidenced a greater level of adherence, whereas having a family history of minor depression was associated with a lower adherence. The amount of therapist time spent per module was associated with a lower level of adherence. Finally, having more ADHD-like symptoms, as measured with the ASRS, was associated with lower adherence, as was being male.

**Table 7:** Final model of predictors of treatment adherence.

	Level of adherence	
	*b* (SE)^a^	*p*
Predictor		
Male gender	-.42 (.14)	**
Level of education	.17 (.14)	
Family history of social anxiety	.35 (.14)	*
Family history of depression	-.25 (.15)	
Family history of minor depression	-.28 (.14)	*
ASRS	-.56 (.14)	***
Treatment credibility	.72 (.15)	***
Therapist time (min) per module	-1.01 (.14)	***

Note. Significant effects on outcome are denoted as:

***, *p* < ∙001;

**, *p* < ∙01

and *, *p* < ∙05.

^a^ Values represent standardized beta coefficients predicting treatment adherence (operationalized as number of modules) at post-treatment. Positive values indicate higher estimated adherence.

## Discussion

The aim of the present study was to identify predictors of symptomatic improvement and adherence to ICBT for SAD within the context of routine psychiatric outpatient care. On average, patients improved at a rate of 20 points on the LSAS-SR during the treatment. Although this demonstrates significant symptomatic improvements, it is worth noting that the average post-treatment LSAS-SR score was approximately 50 (see Table 5), which is well over the LSAS-SR total score ≤ 30 criteria for remission from SAD as proposed by Mennin [46] and Rytwinski [47]. This indicates that most patients still had at least mild SAD after treatment.

Treatment credibility and adherence emerged as significant predictors of a faster rate of improvement during the course of treatment, whereas those patients presenting with a higher overall level of functioning prior to treatment experienced a slower rate of improvement, possibly reflecting a floor effect. Further, treatment credibility and having earlier experience of SAD-like symptoms within the family was associated with increased treatment adherence, whereas ADHD-like symptoms, being male and having a family history of minor depression emerged as factors associated with lower adherence. Finally, the amount of time therapists spent with patients on each treatment module was negatively associated with adherence; however, the most likely explanation for this observation is simply that therapists spent more time with patients who had difficulties complying with the treatment rather than that therapist contact in itself would be a predictor of poor adherence.

Probably the most interesting finding in this study was that patients’ beliefs and expectations towards the treatment seem to be more critical for a successful intervention than clinical or demographic factors. The association between treatment credibility and adherence might indicate that when patients feel confident that the treatment will be helpful, they may be more willing to engage in the treatment, consequently being more exposed to effective components of the CBT protocol (e.g. confronting feared thoughts and situations). Indeed, treatment credibility has previously been identified as a factor that plays a significant role in the effectiveness of ICBT interventions [13,14]. Although a high level of treatment credibility appears to be the most important prognostic factor for adherence, one must be cautious to conclude that this is a causal relationship, given that treatment credibility was not measured until the second week of treatment; it might have been influenced by early response which also seems to be a strong outcome predictor. The relation between early response and outcome of ICBT for SAD has recently been studied in detail elsewhere [48]. One final remark on treatment credibility is that since this study was done on a sample where participants had specifically applied for an ICBT interventions, it is interesting that the effect of treatment credibility still appear to be such a strong predictor. It would be interesting to compare its effect with a sample at a regular primary care clinic where patients have no preference on the delivery modality of CBT.

### Strengths and Limitations

The main benefit of this study was the size of the sample and the routine care setting of treatment delivery. This increases the likelihood of the results being generalizable to similar clinical contexts were ICBT for SAD is being considered for dissemination. Additionally, outcome assessments were administered systematically and frequently and inclusion of patients spanned over several years.

A discussion on the limitations of the study is warranted. Probably the most evident limitation is the lack of a comparison group, which introduces the inability to control for natural temporal trends as a potential explanation for the observed change in SAD symptoms occurring during the course of treatment. This is often a common issue in effectiveness studies in clinical settings. In this case, the aim of the study was to explore differences in treatment outcome within the context of a regular care clinic, effectively evaluating all patients who had received treatment for SAD over the course of a longer time frame. In such a naturalistic setting, there would have been additional ethical considerations of implementing a wait-list condition since there are also operational demands in regard to process lead times and improvement efforts of health care quality (e.g. decreasing time to wait between applying for treatment and receiving adequate care). Also, to measure adherence, we used the number of activated treatment modules, which means that we do not know whether patients actually completed the last activated module or not. This is apparently a limitation in measurement reliability. Since adherence in conventional face-to-face CBT typically refer to both in-session and out of session behaviour, the remote therapist guidance inherent in the ICBT delivery format makes it more challenging to have full insight into the actual (as opposed to reported) level of compliance in each module. Of course, tracking actual homework compliance in face-to-face CBT may be equally challenging, given the dependency on patients’ self-reports of out of session behaviour. Another limitation concerns the heavy reliance on subjective self-report data, since this could potentially introduce response bias. As was reported above, self-reported LSAS scores were systematically higher that clinician ratings. However, such a small mean difference (2.59 points) might not be clinically relevant. Further, clinician- and self-rated versions of the LSAS were found to be highly correlated (see also Fresco et al. [49]), which strengthens the assumed validity of the used self-report measurements. Finally, a note on generalizability of these results is that although the generalizability may be higher than would be the case of an RCT, these results might not apply to all primary care clinics settings, since patients included in this sample have actively sought out this treatment rather than being referred by a GP or primary care. Therefore, it might be argued that patients recruited in internet-based intervention studies may not be representative for the typical routine mental health care patient.

### Clinical Implications

The findings of the present study highlight the importance of not only therapeutic compliance but also patients’ beliefs and expectations about the treatment. This study, then, raises the question of how to promote these factors. For example, could the provision of extra support to identified subgroups at risk of low adherence increase the probability of therapeutic compliance (and ultimately treatment response)? Although patients who showed signs of inactivity and lower adherence generally would receive reminders and supportive messages from their therapist in order to help them to stay engaged in the treatment, it is not clear whether these therapist behaviours significantly increased compliance. [14]. Indeed, the importance of therapist support in ICBT is not yet established [50]. For example, it has been observed in several studies of ICBT that more therapist support is associated with both increased adherence and larger treatment effects [51]. On the other hand, in a study where effectiveness and adherence was compared between guided and unguided ICBT for SAD, it was found that therapist guidance increased the likelihood of completing the treatment and that treatment effects were similar among those who completed the treatment regardless of whether they had received guidance or not [52]. Also, in a study comparing three levels of therapist guidance in ICBT for SAD, it was observed that the treatment was equally effective regardless of the amount of guidance patients received [50]. Although it cannot be concluded from the present study that the amount of time that therapists invest in guidance increases patients’ compliance or response to treatment in general, therapists might still be able to better support those patients seeking treatment for SAD who also presents with ADHD-like symptoms (e.g. by using similar organizational strategies that are used to aid patients with ADHD [53]).

## Conclusions

Prediction research is ultimately a tool to help clinicians make informed decisions prior to treatment. Results from this relatively large clinical sample indicate that expectation of treatment effectiveness and adhering to the treatment are critical factors for treatment success in ICBT for SAD. Clinicians might therefore benefit from early identification of probable “low-responders” and to test added measures that could increase therapeutic compliance and credibility. For example, clearly explaining the rationale for and communicating confidence in the treatment might increase patients’ willingness to engage and consequently increase the likelihood of treatment effectiveness. Also, since having ADHD-like symptoms seems to be associated with somewhat lower adherence, patients presenting with these additional challenges might benefit from extra support when working with CBT components central for treatment success. However, more research is needed in order to determine not only what type of support that would be most useful but also what level of guidance would be optimal in terms of providing effective treatments with efficient use of healthcare resources [54].

In sum, ICBT is an effective treatment for individuals suffering from SAD and high treatment credibility and adherence are strong predictors for greater rate of improvement. With the current dissemination of ICBT in regular care as an innovative and cost-effective way of increasing access to evidence based psychotherapy, the online delivery-format of ICBT may have several advantages over conventional CBT, especially for individuals with more severe social anxiety who might fear face-to-face interactions [6,54]. ICBT may therefore be a preferable treatment option for individuals who might otherwise avoid seeking help.

## Supporting Information

S1 Table Step-wise analyses of predictors of symptomatic change. (DOCX)

S2 Table Step-wise analyses of predictors of treatment adherence. (DOCX)
