# Supplementary material for: Predictors of Symptomatic Change and Adherence in Internet-Based Cognitive Behaviour Therapy for Social Anxiety Disorder in Routine Psychiatric Care
Source: PLoS One. 2015 Apr 20;10(4):e0124258. doi: 10.1371/journal.pone.0124258 (PMC4404057; doi:10.1371/journal.pone.0124258)
Supplement: S1 Table — (DOCX) [file pone.0124258.s001.docx]

**S1 Table. Step-wise analyses of predictors of symptomatic change.**

| **Domain/predictor** | **Change over time** | |  | **Post-treatment score** | |
| --- | --- | --- | --- | --- | --- |
|  | ***b* (SE)^a^** | ***p*** |  | ***b* (SE)^b^** | ***p*** |
| **Socio-demographic variables** | | | | | |
| **Step 1** | | | | | |
| Age | 1.88 (.98) | .056 |  | -1.57 (1.20) | .192 |
| Gender | 1.17 (.81) | .149 |  | .12 (1.00) | .903 |
| Level of education | .81 (.84) | .336 |  | -6.10 (1.03) | .000 |
| Employment | 1.48 (.82) | .070 |  | -2.98 (.96) | .002 |
| Married / cohabiting | -.57 (.85) | .500 |  | -1.68 (1.05) | .111 |
| Have children | -.64 (.99) | .516 |  | -.63 (1.22) | .605 |
| **Step 2 (retain effects at *p* < .20)** ^c^ | | | | | |
| Age | 1.84 (.79) | .020 |  | -2.90 (.99) | .004 |
| Gender | 1.09 (.78) | .161 |  | .40 (.99) | .684 |
| Employment | 1.61 (.79) | .042 |  | -4.06 (.95) | .000 |
| **Steps 3 and 4 (retain effects at *p* < .10 and *p* < .05. respectively)** | | | | | |
| Age | 1.83 (.79) | .021 |  | -2.90 (.99) | .004 |
| Employment | 1.59 (.79) | .045 |  | -4.05 (.95) | .000 |
|  |  |  |  |  |  |
| **Family history** **of mental illness** | | | | | |
| **Step 1** | | | | | |
| Family history of social anxiety disorder | -.10 (.79) | .904 |  | 1.64 (1.01) | .104 |
| Family history of social anxiety disorder-like symptoms | -.10 (.78) | .898 |  | -.54 (.99) | .585 |
| Family history of anxiety | -.97 (.80) | .224 |  | -2.67 (1.01) | .008 |
| Family history of depression | -.03 (.81) | .968 |  | .47 (1.03) | .649 |
| Family history of minor depression | .68 (.80) | .398 |  | 2.40 (1.01) | .018 |
| Family history of panic disorder | 1.33 (.79) | .091 |  | .32 (1.00) | .748 |
| Family history of neuropsychiatric condition | .74 (.80) | .357 |  | 1.27 (1.02) | .210 |
| Family history of psychosis | -.53 (.79) | .501 |  | .28 (1.00) | .778 |
| Family history of bipolar disorder | -.40 (.79) | .617 |  | .06 (1.01) | .951 |
| Family history of dependence / substance abuse | -.92 (.79) | .245 |  | -.92 (1.01) | .364 |
| Family history of suicide attempts | .58 (.82) | .475 |  | 1.35 (1.03) | .192 |
| Family history of suicide completed | 1.14 (.80) | .153 |  | .02 (1.01) | .987 |
| **Steps 2 and 3 (retain effects at *p* < .2 and *p* < .10 respectively)** | | | | | |
| Family history of panic disorder | 1.35 (.78) | .084 |  | .53 (1.00) | .594 |
|  |  |  |  |  |  |
| **Clinical characteristics** |  |  |  |  |  |
| **Step 1** | | | | | |
| CGI-S | 1.42 (2.60) | .584 |  | 9.95 (3.05) | .001 |
| GAF | 5.91 (2.44) | .017 |  | 1.42 (2.85) | .619 |
| Comorbidity | -1.59 (2.10) | .451 |  | -3.39 (2.45) | .168 |
| MADRS-S | 2.22 (2.41) | .357 |  | 5.41 (2.80) | .055 |
| ASRS | -.91 (2.05) | .657 |  | 4.90 (2.42) | .044 |
| AUDIT | -.20 (1.77) | .909 |  | -2.39 (2.08) | .253 |
| DUDIT | -3.71 (3.17) | .244 |  | -6.05 (3.63) | .098 |
| Years since onset of symptoms | -.20 (2.13) | .926 |  | -2.45 (2.50) | .329 |
| Age of onset of symptoms | .36 (2.02) | .858 |  | -2.45 (2.38) | .305 |
| General self-efficacy | -.76 (2.19) | .729 |  | -6.33 (2.59) | .016 |
| Concurrent psychotropic medication | .27 (1.81) | .882 |  | 2.54 (2.13) | .236 |
| History of depression | 1.93 (1.96) | .326 |  | .70 (2.29) | .760 |
| History of inpatient psychiatric care | -2.24 (1.84) | .226 |  | -3.54 (2.16) | .104 |
| Attempted suicide | -1.92 (1.80) | .288 |  | 3.28 (2.12) | .124 |
| **Steps 2, 3 and 4 (retain effects at *p* < .2. *p* < .10 and *p* < .05. respectively)** | | | | | |
| GAF | 3.46 (.86) | .000 |  | -8.86 (1.04) | 000 |
|  |  |  |  |  |  |
| **Treatment-related factors** | | | | | |
| **Step 1** | | | | | |
| Adherence | -4.06 (1.34) | .002 |  | -5.91 (1.71) | .001 |
| Treatment credibility | -4.38 (.85) | .000 |  | -3.17 (1.07) | .003 |
| Therapist time | .73 (1.19) | .541 |  | 2.34 (1.53) | .127 |
| Patient logged time online | -.28 (.94) | .768 |  | 4.28 (1.22) | .000 |
| Patient number of logins | -.20 (1.66) | .903 |  | 4.19 (2.14) | .051 |
| Patient number of mouse clicks | .07 (1.75) | .969 |  | -1.82 (2.26) | .419 |
| Patient number of sent messages | -.37 (1.83) | .844 |  | -6.57 (2.34) | .005 |
| Patient number of posted messages on forum | 1.02 (1.89) | .275 |  | -1.12 (2.41) | .350 |
| **Steps 2, 3 and 4 (retain effects at *p* < .2. *p* < .10 and *p* < .05. respectively)** | | | | | |
| Adherence | -4.13 (.93) | .000 |  | -6.41 (1.19) | .000 |
| Treatment credibility | -4.44 (.83) | .000 |  | -2.95 (1.08) | .006 |

^a^ Values represent standardized beta coefficients predicting the rate of change (slope) in self-rated Liebowitz Social Anxiety Scale (LSAS-SR) scores over assessment occasions. Coefficients therefore represent the interaction of the predictor with time. Negative values indicate greater change during treatment.

^b^ Values represent standardized beta coefficients predicting LSAS-SR at post-treatment. Negative values indicate lower estimated post-treatment scores.
