# Supplementary material for: Predictors of Symptomatic Change and Adherence in Internet-Based Cognitive Behaviour Therapy for Social Anxiety Disorder in Routine Psychiatric Care
Source: PLoS One. 2015 Apr 20;10(4):e0124258. doi: 10.1371/journal.pone.0124258 (PMC4404057; doi:10.1371/journal.pone.0124258)
Supplement: S2 Table — (DOCX) [file pone.0124258.s002.docx]

**S2 Table. Step-wise analyses of predictors of treatment adherence.**

| **Domain/predictor** | **Level of adherence** | |
| --- | --- | --- |
|  | ***b* (SE)^a^** | ***p*** |
| **Socio-demographic variables** |  |  |
| **Step 1** | | |
| Age | .16 (.15) | .278 |
| Male gender | -.53 (.12) | .000 |
| Level of education | .32 (.13) | .012 |
| Employment | .10 (.13) | .411 |
| Married / cohabiting | .21 (.13) | .108 |
| Have children | -.07 (.15) | .627 |
| **Step 2 (retain effects at *p* < .20)** |  |  |
| Male gender | -.53 (.12) | .000 |
| Education | .37 (.12) | .003 |
| Married / cohabiting | .20 (.12) | .112 |
| **Steps 3 and 4 (retain effects at *p* < .10 and *p* < .05, respectively)** | | |
| Male gender | -.54 (.12) | .000 |
| Education | .40 (.12) | .001 |
|  |  |  |
| **Family history of mental illness** |  |  |
| **Step 1** |  |  |
| Family history of social anxiety disorder | .00 (.12) | .982 |
| Family history of social anxiety disorder-like symptoms | .27 (.12) | .024 |
| Family history of anxiety | -.15 (.12) | .228 |
| Family history of depression | -.25 (.12) | .049 |
| Family history of minor depression | -.28 (.12) | .020 |
| Family history of panic disorder | .08 (.12) | .506 |
| Family history of neuropsychiatric condition | -.12 (.12) | .348 |
| Family history of psychosis | .09 (.12) | .484 |
| Family history of bipolar disorder | .10 (.12) | .434 |
| Family history of dependence / substance abuse | -.03 (.12) | .791 |
| Family history of suicide attempts | -.14 (.12) | .270 |
| Family history of suicide completed | -.06 (.12) | .632 |
| **Steps 2, 3 and 4 (retain effects at *p* < .20,  *p* < .10 and *p* < .05, respectively)** | | |
| Family history of social anxiety disorder-like symptoms | .27 (.12) | .024 |
| Family history of depression | -.30 (.12) | .015 |
| Family history of minor depression | -.29 (.12) | .016 |
|  |  |  |
| **Clinical characteristics** |  |  |
| **Step 1** | | |
| CGI-S | -.03 (.38) | .942 |
| GAF | -.20 (.35) | .574 |
| Comorbidity | -.01 (.30) | .971 |
| MADRS-S | -.26 (.34) | .455 |
| ASRS | -.87 (.30) | .005 |
| AUDIT | -.31 (.26) | .232 |
| DUDIT | -.24 (.44) | .586 |
| Years since onset of symptoms | .35 (.31) | .255 |
| Age of onset of symptoms | -.25 (.30) | .399 |
| General self-efficacy | -.06 (.32) | .858 |
| Concurrent psychotropic medication | -.36 (.27) | .176 |
| History of depression | -.22 (.29) | .439 |
| History of inpatient psychiatric care | -.08 (.27) | .764 |
| Attempted suicide | -.15 (.26) | .575 |
| **Step 2 (retain effects at *p* < .20)** | | |
| ASRS | -.98 (.23) | .000 |
| Concurrent psychotropic medication | -.37 (.24) | .127 |
| **Steps 3 and 4 (retain effects at *p* < .10 and *p* < .05, respectively)** | | |
| ASRS | -.80 (.16) | .000 |
|  |  |  |
| **Treatment-related factors** |  |  |
| **Step 1, 2, 3 and 4 (retain effects at *p* < .20,  *p* < .10 and *p* < .05, respectively)** | | |
| Treatment credibility | .59 (.11) | .000 |
| Therapist time (min) per module | -.98 (.11) | .000 |
|  |  |  |

^a^ Values represent standardized beta coefficients predicting treatment adherence (operationalized as number of modules) at post-treatment. Positive values indicate higher estimated adherence.
